# Limited expression of non-integrating CpG-free plasmid is associated with increased nucleosome enrichment

**DOI:** 10.1371/journal.pone.0244386

**Published:** 2020-12-21

**Authors:** Omar Habib, Rozita Mohd Sakri, Nadiah Ghazalli, De-Ming Chau, King-Hwa Ling, Syahril Abdullah

**Affiliations:** 1 Department of Biomedical Science, Faculty of Medicine and Health Sciences, Universiti Putra Malaysia (UPM), Serdang, Selangor, Malaysia; 2 Genetics and Regenerative Medicine Research Centre, Faculty of Medicine and Health Sciences, Universiti Putra Malaysia (UPM), Serdang, Selangor, Malaysia; 3 Faculty of Applied Sciences, Universiti Teknologi MARA (UiTM), Shah Alam, Selangor, Malaysia; 4 UPM-MAKNA Cancer Research Laboratory, Institute of Bioscience, Universiti Putra Malaysia (UPM), Serdang, Selangor, Malaysia; University of Pennsylvania, UNITED STATES

## Abstract

CpG-free pDNA was reported to facilitate sustained transgene expression with minimal inflammation *in vivo* as compared to CpG-containing pDNA. However, the expression potential and impact of CpG-free pDNA in *in vitro* model have never been described. Hence, in this study, we analyzed the transgene expression profiles of CpG-free pDNA *in vitro* to determine the influence of CpG depletion from the transgene. We found that in contrast to the published *in vivo* studies, CpG-free pDNA expressed a significantly lower level of luciferase than CpG-rich pDNA in several human cell lines. By comparing novel CpG-free pDNA carrying CpG-free *GFP* (pZGFP: 0 CpG) to CpG-rich *GFP* (pRGFP: 60 CpGs), we further showed that the discrepancy was not influenced by external factors such as gene transfer agent, cell species, cell type, and cytotoxicity. Moreover, pZGFP exhibited reduced expression despite having equal gene dosage as pRGFP. Analysis of mRNA distribution revealed that the mRNA export of pZGFP and pRGFP was similar; however, the steady state mRNA level of pZGFP was significantly lower. Upon further investigation, we found that the CpG-free transgene in non-integrating CpG-free pDNA backbone acquired increased nucleosome enrichment as compared with CpG-rich transgene, which may explain the observed reduced level of steady state mRNA. Our findings suggest that nucleosome enrichment could regulate non-integrating CpG-free pDNA expression and has implications on pDNA design.

## Introduction

Plasmid DNA (pDNA) has played an essential role in advancing basic research, biotechnology and medicine. Due to its ability to facilitate transgene expression, it becomes practical to express, silence, augment the levels of a gene(s) of interest, or even introduce foreign gene(s) [1]. This allows better insight into the biology of a system and consequently offers the ability to control and manipulate such a system for beneficial purposes such as gene therapy. As a result, pDNA has undergone various improvements to better facilitate transgene expression. Some of the improvements are the incorporation of novel promoters, introns, and chromatin insulators [2, 3] in the pDNA design. One of the more noteworthy improvements is the development of CpG (cytosine-guanine dinucleotides)-depleted pDNA or commonly known as CpG-free pDNA, where CpG dinucleotides are completely depleted from the whole pDNA, including the transgene.

CpGs in pDNA of bacterial origin differ from CpGs in vertebrate DNA in the frequency and methylation status. CpGs exist more frequently in bacterial pDNA (1 in 16 dinucleotides compared to 1 in 64 dinucleotides in vertebrates DNA) and are not methylated. In contrast, 60–90% of CpGs in vertebrate DNA are methylated [4]. This distinction is crucial in antigen recognition for immune defense. Toll-like Receptor 9 (TLR9) recognizes non-methylated CpG-containing DNA fragments to be foreign, and will accordingly induce an acute inflammatory response [5]. Even the presence of a single CpG in pDNA was enough to elicit profound inflammation *in vivo* [6]. As a result, CpG-induced inflammation leads to immune-mediated destruction of transgene-carrying cells [7, 8], repression of transgene expression due to the direct action of pro-inflammatory cytokines [9–11], and prolonged inflammation, which could result in the development of autoimmunity [12]. Moreover, studies have shown that the unmethylated CpGs in pDNA were prone to de novo DNA methylation-mediated silencing that resulted in the loss of transgene expression [13–18]. Given these points, removing CpGs from pDNA would be advantageous in an *in vivo* system.

The first CpG-free pDNA developed was able to significantly reduce inflammation, which includes levels of pro-inflammatory cytokines, and neutrophil infiltration indicative of minimal tissue damage, irrespective of topical lung or systemic delivery [6]. Subsequent studies also reported the use of CpG-free pDNA only induced minimal host inflammatory response and was safer than the CpG-containing pDNA [19–21]. In addition, intranasal delivery of CpG-free pDNA into mouse lungs led to a high and sustained luciferase expression up to 28 days post-administration. In comparison, a CpG-containing pDNA expression only lasted for approximately 2 weeks before declining drastically, despite the presence of the pDNA on days where the transgene was silenced [6]. Besides mouse lungs [6, 19, 22, 23], CpG-free pDNA was shown to have a better *in vivo* expression profile than CpG-containing pDNAs regardless of delivery methods and target tissues. These include hydrodynamic injection to mouse liver [24, 25], localized injection to rat corneas [26], and intravenous injection to mouse tumour [27]. These studies demonstrated the benefits of utilizing CpG-free pDNA to achieve higher and prolonged transgene expression *in vivo*. To the best of our knowledge, similar benefits have not been reported in *in vitro* applications.

Studies have shown that the presence of CpG often hinders transgene expression due to DNA methylation [14, 15, 28]. The elimination of CpG can potentially circumvent the issue. However, one of the major challenges is to construct the CpG-free pDNA, namely the depletion of CpG from the transgene, without changing its regulatory binding sites and coding sequence. As described earlier, even though vertebrate DNA has lower CpG frequency as compared to bacterial pDNA, the presence of CpG in the vertebrate coding gene is unavoidable [4]. Depleting CpG from vertebrate-derived transgene requires accurate and detailed gene optimization strategies, which include exclusion of AU repeat elements, UpA dinucleotides, RNA secondary structure, and cryptic splice sites. The resulting CpG-free transgene requires codon optimization to remove any rare codon that would negatively impact transgene expression [29]. Given the arduous steps involved in de novo transgene construction, the advantages of utilizing CpG-free pDNA (CpG-free backbone and transgene) as compared to CpG-free pDNA backbone with CpG-containing transgene in *in vitro* models are unknown. Identifying the pros and cons of either construct, would provide deeper understanding on the limits and potentials of the CpG-free pDNA.

Here, we analyzed the *in vitro* transgene expression from CpG-free pDNA, specifically to determine the influence of CpG depletion from the transgene. First, transgene expression of a CpG-free pDNA was compared to CpG-rich pDNA. These two pDNAs carry luciferase reporter gene but with different pDNA backbone. Next, we constructed second generation of pDNAs with similar 0 CpG backbone but carry either zero CpG *GFP* (pZGFP) or CpG-rich *GFP* (pRGFP). The data obtained shows that the pZGFP exhibited reduced transgene expression when compared to pRGFP in several cell lines, and this was due to a reduced rate of transcription. Notably, the absence of CpG on the pZGFP transgene was associated with an increase of nucleosome density that could limit its expression.

## Materials and methods

### Plasmid DNA (pDNA)

In total, 4 different non-integrating pDNAs were utilized in this study. pCIKLux (kindly provided by the Gene Medicine Research Group, University of Oxford, UK) and pCpGfree-Lucia (InvivoGen, USA) each carry a luciferase reporter gene, were utilized in Section 3.1. Henceforth, these plasmids will be referred to as pCpG-rich (pCIKLux) and pCpG-free (pCpGfree-Lucia). In addition, pZGFP and pRGFP pDNAs were constructed from the pCpGfree-Lucia pDNA backbone, after excising the luciferase gene with *NcoI* and *NheI* restriction enzymes and replaced with green fluorescent protein (*GFP*) genes, specifically the EmGFP variants. The CpG-free *GFP* (ZGFP) transgene was amplified by PCR from pMOD-ZGFP::Sh (InvivoGen, USA) using the forward (5’-GAGGGCCA**CCATGG**TTTCTAA-3’) and reverse (GCAAGCTAGCTTACTTGTAC AGCTCATCCATTCCCAGAG) primers designed with restriction enzyme sites to complement the pCpG-free backbone (**bold**: *NcoI*; underlined: *NheI*). As for CpG-rich *GFP* (RGFP) transgene, pLenti6.3-V5-GW-EmGFP (Thermo Fisher Scientific, USA) was used as PCR template and amplified using the forward (5’-AAT**CCATGG**T GAGCAAGGGCGAGGAGCTGTTCAC-3’) and reverse (5’- CAAGCTAGCTTACTTG TACAGCTCGTCCATGCCGA-3’) primers. The resulting pZGFP and pRGFP constructs were confirmed to be mutation-free by Sanger sequencing (First BASE Laboratories Sdn. Bhd., Malaysia). Peptide and DNA sequence alignment were performed using NCBI’s Needleman-Wunsch Global Protein Sequence Alignment web service (http://blast.ncbi.nlm.nih.gov/Blast.cgi) and NCBI’s Needleman-Wunsch Global Nucleotide Sequence Alignment web service (http://blast.ncbi.nlm.nih.gov/Blast.cgi) to determine the percentage (%) of identity.

### Cell culture

HEK-293FT, H1299, MCF7, SH-SY5Y, and NIH-3T3 were cultivated in high glucose Dulbecco’s Modified Eagle’s Medium (DMEM) with L-glutamine, pyridoxine hydrochloride, and 110 mg/L sodium pyruvate, supplemented with 10% Fetal Bovine Serum (FBS), 1% penicillin-streptomycin (10,000 U/mL), and 1.5 g/L sodium bicarbonate (Thermo Fisher Scientific, USA) at pH 7.4. The cells were incubated and maintained in an incubator with 5% CO_2_ humidified atmosphere at 37°C. Only cells with passage number of 30 or below were utilized to avoid any unwanted effects due to repeated cell passaging. As for the primary mouse fibroblast cells, the fibroblast isolation protocol was adapted and modified from Takahashi et al. (2007) [30]. The mouse tail fibroblast was isolated post-mortem from 4 weeks old wildtype C57BL/6J mouse strain. Briefly, the tail was excised; the superficial dermis was peeled and rinsed twice with 70% ethanol, followed by 1x Phosphate Buffered Saline (PBS) pH 7.4. The tail biopsies were minced into roughly 1cm pieces, placed on 0.1% gelatin-coated culture dishes, and incubated in DMEM supplemented with 10% FBS, antibiotics (50 U/mL penicillin and 50 μg/mL streptomycin), 4 mM L-glutamine, and 0.1 mM non-essential amino acids (NEAA) (Thermo Fisher Scientific, USA). The culture dishes were incubated at 37°C with 5% CO_2_. Cells that migrated out from the minced tissue pieces were transferred to new 0.1% gelatin-coated culture dishes (passage 1) and maintained in DMEM containing 10% FBS. In this study, primary tail fibroblasts were utilized within three passages to avoid replicative senescence.

### Lipid and Polymer gene transfer agent (GTA) formulation and cell transfection

The cationic lipid-based gene transfer agent (GTA) used in this study was the Lipofectamine™ 2000 (Thermo Fisher Scientific, USA) and henceforth will be referred to as “Lipid GTA”. The lipid/pDNA complexes were prepared following the manufacturer’s protocol. The ratio of pDNA (in μg): lipid GTA (in μL) utilized was 1:3, which is the optimal ratio as reported by the manufacturer. The cationic polymer-based GTA used in this study was the branched polyethylenimine (bPEI) with molecular weight of 25 kDa (Sigma-Aldrich, USA), and henceforth will be referred to as “Polymer GTA”. The ratio of pDNA (in μg): polymer GTA (in μg) utilized was 1:1.29, which corresponds to polymer nitrogen to DNA phosphate (N:P) molar ratio of 10:1 that is optimal for most cells [20, 22, 31]. For cell transfection, cell-seeding density used was 2.5 x 10^4^ cells/cm^2^ and 1 μg of pDNA was utilized to transfect every 5.0 x 10^4^ cells. The amount of pDNA and cell-seeding density were scaled accordingly to accommodate tissue-culture flask area (cm^2^). Typically, cells were seeded and allowed to adhere for 24 hours. Next, cells were incubated in Lipid or Polymer GTA formulation for 5 hours before the formulation was discarded. Following this, the cells were cultivated in the appropriate growth medium until selected time-point(s).

### Luciferase assay

HEK-293FT, H1299, MCF7, and SH-SY5Y cells were seeded at a density of 5.0 x 10^4^ cells/well in 24-well plate and transfected with 1 μg of pCpG-free or pCpG-rich complexed with Lipid GTA. Harvesting of cell lysates and quantification of expressed luciferase was performed using the Luciferase Assay System Kit (Promega, USA) and GloMax^TM^ 20/20 luminometer (Promega, USA), according to manufacturer’s protocol. The amount of total protein from the cell lysates was determined based on a colorimetric method using the Bio-Rad DC Protein Assay (Bio-Rad, USA) and was utilized to normalize the luciferase expression. The expression was presented as mean relative light unit (RLU) per milligram of total protein (RLU/mg protein). Cell lysate from non-transfected cells was used as a control to exclude any background signal.

### GFP protein expression analysis by flow cytometry

HEK-293FT, NIH-3T3, and primary mouse fibroblast cells were seeded at a density of 1.0 x 10^5^ cells/well in 12-well plate and transfected with 2 μg of pZGFP or pRGFP, complexed with Lipid or Polymer GTA. At selected time-points, adhered cells were dissociated and resuspended in PBS with 1% of FBS (Thermo Fisher Scientific, USA). The GFP expression was analyzed using BD LSRFortessa™ (BD Biosciences, USA) flow cytometer, and non-transfected cells were used for gating purposes to exclude clumped cells and as a reference for GFP^-^ cells. The minimum total event was set to 10,000. Parameters assessed were the % of GFP^+^ cells (indicates the fraction of cells expressing GFP from the gated single cell population) and mean fluorescence intensity (MFI) (indicates the average GFP intensity in a single cell from the GFP^+^ cell population).

### Cell viability analysis by MTT assay

HEK-293FT cells were seeded at a density of 1.5 x 10^4^ cells/well in a 96-well plate. For transfection, the cells in each well were transfected with 0.6 μg pZGFP or pRGFP complexed with 1.2 μl of Lipid GTA. At Day 1 and 2 post-transfection, 20 μL of MTT (Amresco, USA) was added to the growth medium in each well. The cells were incubated at 37°C in a humidified atmosphere for an additional 4 hours. Excess dye was then removed, and insoluble formazan crystals were dissolved in 100 μL dimethyl sulfoxide (DMSO) (Sigma-Aldrich, USA). Spectrophotometer readings were measured using a microplate reader (ASYS Hitech, Austria) at a wavelength of 570 nm, with 690 nm as the reference filter. Non-treated cells were used as negative control. Additional controls for this study were (i) cells treated with 1.2 μL of Lipid GTA only, and (ii) cells transfected with 0.6 μg of the circularized pCpG-free backbone (devoid of transgene) complexed with 0.9 μL of Lipid GTA. Non-treated cells were used as a control with 100% viability. The data are presented as relative cell viability (%) as compared with non-treated cells.

### Gene dosage by absolute qPCR

HEK-293FT cells were seeded at a density of 5.0 x 10^4^ cells/well in 24-well plate and transfected with 1 μg of pCpG-free or pCpG-rich, complexed with Lipid GTA. Total DNA was extracted from cells at Day 1 post-transfection using the DNeasy Blood and Tissue Kit (Qiagen, Germany), according to the manufacturer’s protocol. Only 30 ng of extracted DNA was used as qPCR template. The pDNA copy number in samples were determined through absolute qPCR using standard curves generated with known pDNA copy numbers. The standard curves were generated using purified pZGFP and pRGFP plasmids at 1:10 serial dilution ranging between 210 ng and 2.1 pg.

Absolute qPCR was performed using the LightCycler^®^ 480 Real-Time PCR System Instrument (Roche Diagnostics, Switzerland) [32]. Data generated were analyzed and quantified with the LightCycler^®^ 480 Software version 1.5 (Roche Diagnostics, Switzerland), employing the second derivative maximum method [33] to calculate the Cp (Crossing Point) value. To increase specificity of the assay, Universal Probe Library (UPL) hydrolysis probes that bind specifically to the transgene were utilized. qPCR was performed in a final reaction volume of 10 μL, containing 1X LC480 Probe Master Mix (Roche Diagnostics, Switzerland), 0.1 μM of UPL Probe (Roche Diagnostics, Switzerland), 1 μM of each forward and reverse primer, DNA template, and appropriate volume of nuclease-free water. The qPCR program utilized was as follows: initial denaturation of 95°C for 10 minutes, followed by 45 cycles of 95°C for 10 seconds, 60°C for 30 seconds and 72°C for 8 seconds, and finally 40°C for 30 seconds. Primer pairs and UPL probes are: *ZGFP* (forward–CTGCTCTGTCCAAAGACCCTA, reverse–AATTCCTGCTGCTGTCACAA, UPL Probe# - 79, and amplicon size– 74 bp) and *RGFP* (forward–TGGTCCTGCTGGAGTTCG, reverse–CTTGTACAGCTCGTCCATGC, UPL Probe# - 70, and amplicon size– 62 bp).

### mRNA distribution analysis by absolute qPCR

HEK-293FT cells were seeded at a density of 1.0 x 10^5^ cells/well in 12-well plate and transfected with 2 μg of pZGFP or pRGFP, complexed with Lipid GTA. Nuclear and cytoplasmic mRNAs were extracted from cells at Day 1 post-transfection using the Cytoplasmic and Nuclear RNA Purification Kit (Norgen Biotek Corp., Canada), according to manufacturer’s protocol. Five hundred (500) ng of extracted RNAs were reverse transcribed to cDNA using Transcriptor First Strand cDNA Synthesis Kit (Roche Diagnostics, Switzerland) using both anchored-oligo(dT)_18_ and random hexamer primers. Next, the cDNAs were diluted 10x using nuclease-free water (Roche Diagnostics, Switzerland). Absolute qPCR was performed using 1 μl of 10x-diluted cDNAs to quantify mRNA copy number in nuclear and cytoplasmic fractions [34] as described in Section 2.7.

### Formaldehyde-Assisted Isolation of Regulatory Element (FAIRE)

FAIRE analysis was performed based on an established protocol [35]. HEK-293FT cells were seeded at a density of 6.25 x 10^5^ cells/well in T25 flask and transfected with 12.5 μg of pZGFP or pRGFP complexed with Lipid GTA. At Day 1 post-transfection, cells were dissociated, fixed in suspension with 1% formaldehyde solution for 9 minutes at room temperature, and subsequently quenched with glycine (125 mM). Cells were pelleted by centrifugation at 850 x g for 5 minutes in 4°C and washed twice with PBS. The cells were then resuspended in lysis buffer (2% Triton X-100, 1% SDS, 100 mM NaCl, 10 mM Tris–HCl, pH 8.0, 1 mM EDTA, 1x protease inhibitor cocktail (Roche Diagnostics, Switzerland)). Sonication was performed on the samples using Bioruptor Plus sonicator (Diagenode, Belgium) at high power for 15 cycles (15 seconds on; 15 seconds off) to obtain DNA fragments ranging in 200–500 bp. Twenty-five (25)% of the sonicated samples was reverse cross-linked by adding 0.5 μg/μl proteinase K (Nacalai Tesque, Japan) and incubated at 65°C for 6 hours. This fraction was used as “Input” fraction because histone proteins would be eliminated during reverse cross-linking. The remaining 75% of the sample was not reverse cross-linked, and this fraction was used as “FAIRE” fraction.

Next, DNA purification was performed on both “Input” and “FAIRE” fractions by adding an equal volume of phenol: chloroform: isoamyl alcohol (25:24:1)(Nacalai Tesque) and vortexed for 10 seconds. The samples were centrifuged at high speed for 10 minutes at room temperature to obtain the aqueous phase. The aqueous phase was transferred, and potassium acetate (3 M, pH 5.5) was added at 1/10 of the sample volume and vortexed for 5 seconds. About 2x volumes of ice-cold 100% ethanol was added, vortexed for 10 seconds, and incubated at -80°C for 2 hours. Samples were centrifuged at high speed for 5 minutes, and supernatants were discarded. Five hundred (500) μl of 70% ethanol was used to resuspend the DNA pellet and centrifuged at high speed for 5 minutes to discard the supernatant. The DNA pellet was air-dried for 15 minutes and resuspended in an appropriate volume of nuclease-free water to make the final DNA concentration of 35 ng/μl.

qPCR was performed in a final reaction volume of 10 μL, containing 1X LightCycler^®^ 480 SYBR Green I Master (Roche Diagnostics, Switzerland), 1 μM of each forward and reverse primer, 35 ng of the sample, and appropriate volume of nuclease-free water. The following qPCR program was utilized: initial denaturation of 95°C for 10 minutes followed by 45 cycles of 95°C for 10 seconds, 60°C for 30 seconds and 72°C for 8 seconds, and finally 40°C for 30 seconds. Primers were designed to cover the *hEF1α* promoter region on the pDNA. Additional primers were designed to amplify the 5’ region, middle region, and 3’ region of the transgenes. Primers were also designed to cover the *GAPDH* and *HSAT2* regions as internal controls. Primer pairs utilized were: *GAPDH* (forward—CCCGTCCTTGACTCCCTAGT, reverse—GTGATCGGTGCTGGTTCC, amplicon size—64 bp), *HSAT*2 (forward—GGTGTGATCTCTGCTCGCTAC, reverse—CTGCAATCTCGGCACGTT, amplicon size—74 bp), *hEF1α* (forward—GCAATTGAACTGGTGCCTAGA, reverse—AAGGTGGAGCCAGTACACCA, amplicon size—75 bp), *5’ RGFP* (forward—CCTGAAGTTCATCTGCACCA, reverse—GTAGGTGAAGGTGGTCACGAG, amplicon size—70 bp), *Mid RGFP* (forward—GAGGACGGCAACATCCTG, reverse—CGGCGGTGATATAGACCTTG, amplicon size—70 bp), *3’ RGFP* (forward—TGGTCCTGCTGGAGTTCG, reverse—CTTGTACAGCTCGTCCATGC, amplicon size—62 bp), *5’ ZGFP* (forward—GGTGTTGTCCCAATTCTGGT, reverse—CACAGAGAATTTGTGGCCATT, amplicon size—60 bp), *Mid ZGFP* (forward—AGGTTATGTTCAGGAGAGGACAA, reverse—CTGTTAACCAGTGTATCACCTTCAA, amplicon size—96 bp), *3’ ZGFP* (forward—CTGCTCTGTCCAAAGACCCTA, reverse—AATTCCTGCTGCTGTCACAA, amplicon size—74 bp). Data were analyzed using the 2^-ΔΔCT^ method, where *GAPDH* was used as housekeeping control. Instead of presenting the results as nuclesome-depletion, nucleosome enrichment was determined by modifying the formula to 2^-((ΔInput)–(ΔFAIRE))^ and values were plotted as relative to *GAPDH* nucleosome enrichment.

### Statistical analysis

Data are presented as mean ± standard error of the mean (SEM). Parametric Student’s t-test was applied to compare two unpaired groups, which are the pZGFP and pRGFP, and data were considered to be significantly different when *P* < 0.05. Statistical analyses were performed using GraphPad Prism software v5 (GraphPad, USA). All experiments were repeated at least twice.

## Results

### CpG-free pDNA exhibited limited transgene expression in human cell lines

**Fig 1:**
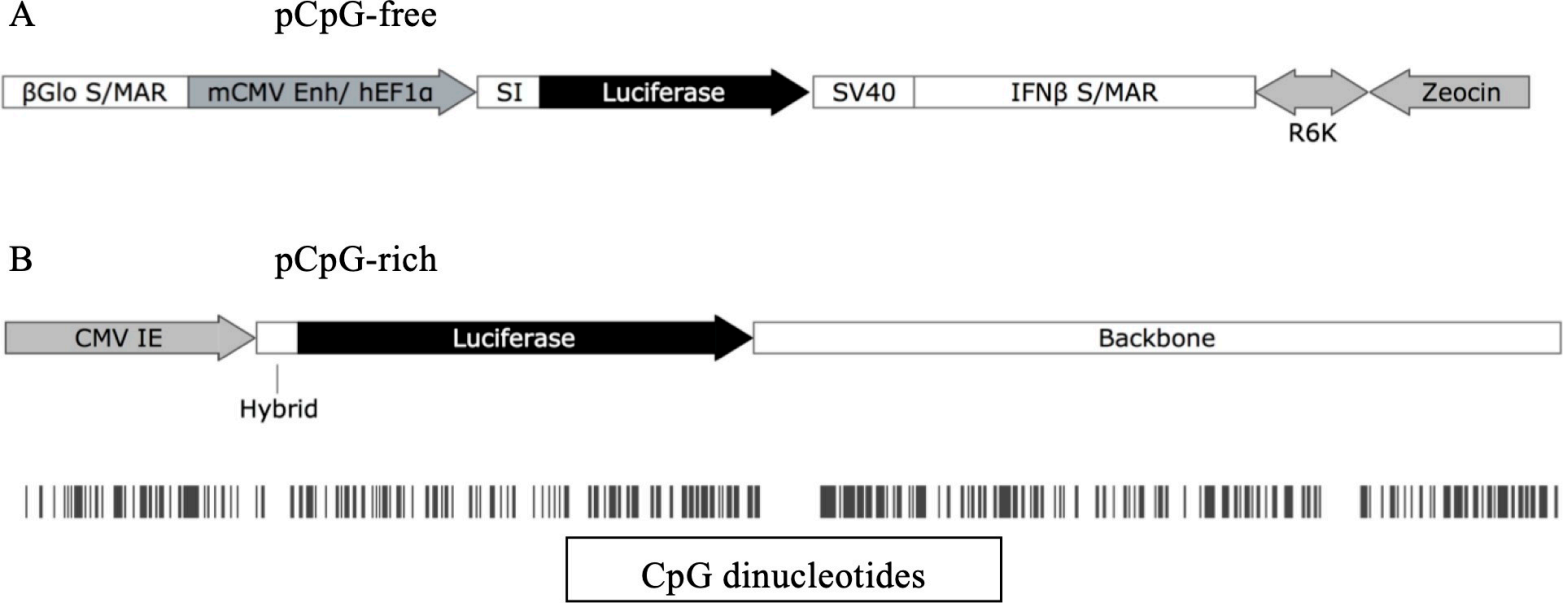
pDNA map and CpG number of luciferase-expressing pDNAs. (A) Size of pCpG-free is 3652 bp without any CpG (0 CpG). The expression of luciferase is under the control of mouse CMV enhancer (mCMV Enh) coupled with human Elongation Factor 1 α (hEF1α). (B) The size of pCpG-rich is 5632 bp with the total CpG number of 317 CpGs (vertical black bars). The cytomegalovirus (CMV) immediate early (IE) promoter has 39 CpGs, and luciferase reporter gene has 96 CpGs. The maps were illustrated using SnapGene software (SnapGene, USA).

First, we sought to recapitulate the prominent *in vivo* expression of CpG-free pDNA, as compared with CpG-rich [6, 22, 24–27], in *in vitro* model (*i*.*e*. human cell lines). This was to serve as a benchmark for the level and duration of CpG-free pDNA expression for subsequent *in vitro* experiments. This was done by utilizing the identical pDNAs reported previously, which are the CpG-free (0 CpG) and CpG-rich (317 CpGs) pDNAs, both expressing the luciferase reporter gene. The transgene in pCpG-rich was driven by the human immediate early cytomegalovirus (hCMV) promoter, which is a typical constitutive viral-based promoter, while the pCpG-free uses a eukaryotic-origin human Elongation Factor 1 α (hEF1α) promoter. The *hEF1α* promoter is known to provide higher and persistent transgene expression compared to *hCMV* in both *in vitro* and *in vivo* systems [2]. The pCpG-rich contains 317 CpGs throughout the pDNA, where 42% of the CpGs are confined in promoter and luciferase reporter gene regions (39 and 96 CpGs respectively), whereas the pCpG-free was devoid of any CpG motif (Fig 1). Equal mass of 1μg of each plasmid was transfected: however, molar ratio of pCpG-rich: pCpG-free was 1:1.5 due to the difference in pDNA size (Fig 1).

**Fig 2:**
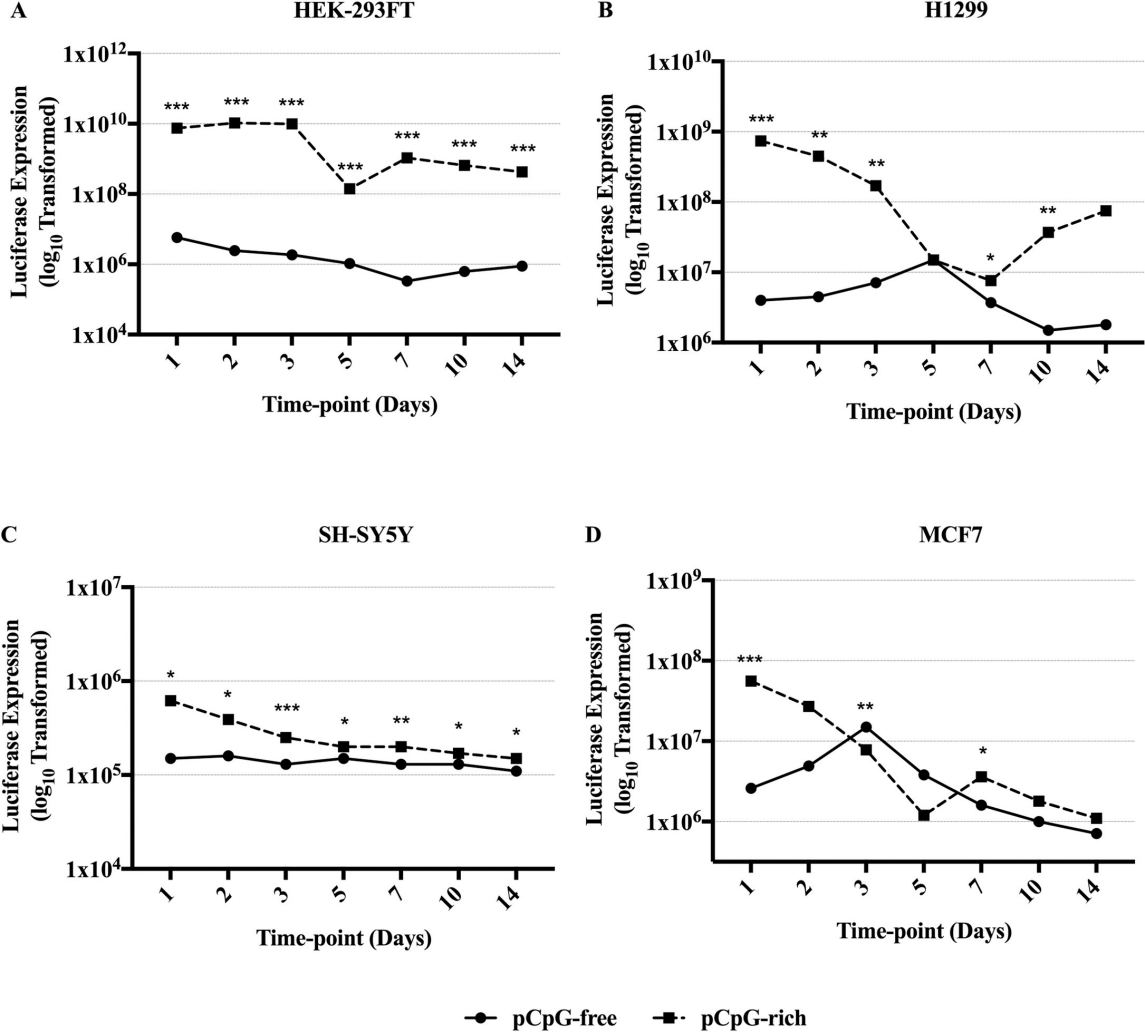
Luciferase reporter gene expression of human cell lines transfected with pCpG-free (solid line) and pCpG-rich (dashed line) complexed with Lipid GTA. (A) HEK-293FT, human embryonic kidney cells. (B) H1299, human non-small cell lung carcinoma. (C) SH-SY5Y, human neuroblastoma cells. (D) MCF7, human breast adenocarcinoma. Luciferase expression was measured at Day 1, 2, 3, 5, 7, 10, and 14 post-transfection and normalized to total protein. Data are presented as log_10_ transformed mean luciferase expression of 3 biological replicates. Asterisk (*) indicates a significant difference for a given time-point between pCpG-free and pCpG-rich determined by Student’s t test (*p<0.05, **p<0.01, ***p<0.001).

In HEK-293FT (Fig 2A), the difference in luciferase expression level was highly significant (p<0.001) from Day 1 where pCpG-free gave ≈ 3 log_10_ lower expression than pCpG-rich. Similarly, in the other cell lines tested, pCpG-free also showed significantly lower luciferase expression than pCpG-rich at Day 1 (Fig 2B–2D) where the differences were ≈ 2 log_10_ in H1299 (p<0.001), ≈ 1 log_10_ in MCF7 (p<0.001) and SH-SY5Y (p = 0.029). The expression of pCpG-free remained low in comparison to pCpG-rich even at Day 14, at ≈ 3 log_10_ lower for HEK-293FT (p<0.001), ≈ 2 log_10_ lower for H1299, ≈ 0.25 log_10_ lower for SH-5YSY (p = 0.025), except for MCF7. Moreover, in HEK-293FT and SH-SY5Y, the luciferase expressions from pCpG-free were consistently and significantly lower across all time points compared to pCpG-rich (Fig 2A and 2C). As for the H1299 and MCF7, the pCpG-free luciferase expressions were observed to fluctuate (Fig 2B and 2D). In general, the luciferase expression pattern from pCpG-free was lower than pCpG-rich in all human cell lines and at most time points, despite higher copy number of pCpG-free transfected. Data indicates usage of CpG-free pDNA over CpG-rich pDNA was not advatageuos in *in vitro* systems.

### Construction of CpG-free pDNAs with variable number of CpG dinucleotides

**Fig 3:**
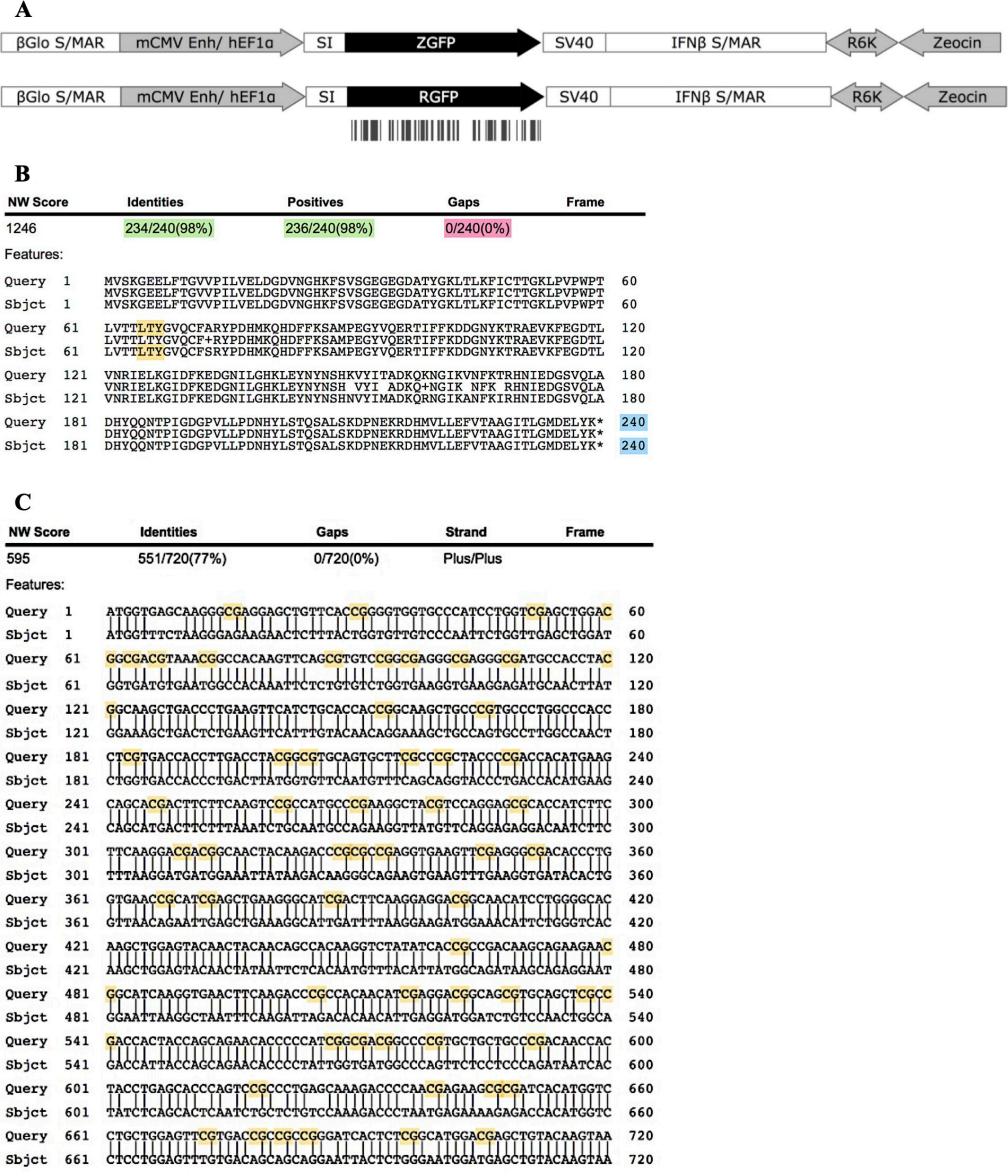
pDNA maps, peptide, and DNA sequences alignment of pZGFP and pRGFP. (A) pZGFP and pRGFP were constructed from the identical CpG-free pDNA backbone. Antibiotic resistance gene is Zeocin™, R6Kγ origin of replication, *ZGFP*, or *RGFP* is under the control of mouse cytomegalovirus (mCMV) coupled with human elongation Factor 1 α (hEF1α). A synthetic intron (SI 126) and simian virus 40 (SV40) polyadenylation signal sequence flank the transgene. Mammalian and bacterial domains were confined by scaffold/matrix attachment regions, from human *IFN-β* (IFNβ S/MAR) and *β-globin* (βGlo S/MAR). The vertical black bars under *RGFP* represent the presence of CpGs. The maps were illustrated using SnapGene software. (B) ZGFP (Sbjct) peptide sequence was aligned to the RGFP peptide sequence (Query). Highlighted in green: % of identity; pink: the presence of gaps; blue: length of peptide sequence; yellow: GFP fluorophore. (C) DNA sequences alignment between *ZGFP* (Sbjct) and *RGFP* (Query). Highlighted in yellow are CpG motifs.

The observed low expression of pCpG-free compared to pCpG-rich may be due to differences in regulatory elements and/or CpG content on transgene. Here, pDNAs with identical CpG-free pDNA backbone (*i*.*e*. regulatory elements) with transgenes of 0 CpG (*ZGFP*) or 60 CpGs (*RGFP*) were constructed to investigate the influence of CpG content on transgene expression. The expression of the transgene (*ZGFP* or *RGFP*) was under the control of mouse cytomegalovirus (*mCMV*) enhancer coupled with human elongation Factor 1α (*hEF1α*) promoter. A synthetic intron (SI 126) and simian virus 40 (SV40) polyadenylation signal were present upstream and downstream of the transgene, respectively, to enhance the transgene expression. In addition, mammalian and bacterial domains were separated by human *IFN-β* and *β-globin* matrix attachment regions (MARs) to avoid interference between the domains that may affect the expression (Fig 3A).

Humanized CpG-depleted (*ZGFP*) and CpG-rich (*RGFP*) *GFP* alleles were utilized, and the proteins encoded by both transgenes were ensured to closely resemble each other. The nucleotide sequences of ZGFP and RGFP were 77% identical, with an equal peptide length of 240 a.a. and 98% protein similarity (Fig 3B and 3C). Importantly, the fluorophore region (Leu_65_-Thr_66_-Tyr_67_) responsible GFP fluorescence [36] was identical between ZGFP and RGFP. This ensures that any discrepancy in fluorescence intensity is not due to differences in amino acid sequences (Fig 3B).

### pZGFP has reduced transgene expression regardless of gene transfer agent

**Fig 4:**
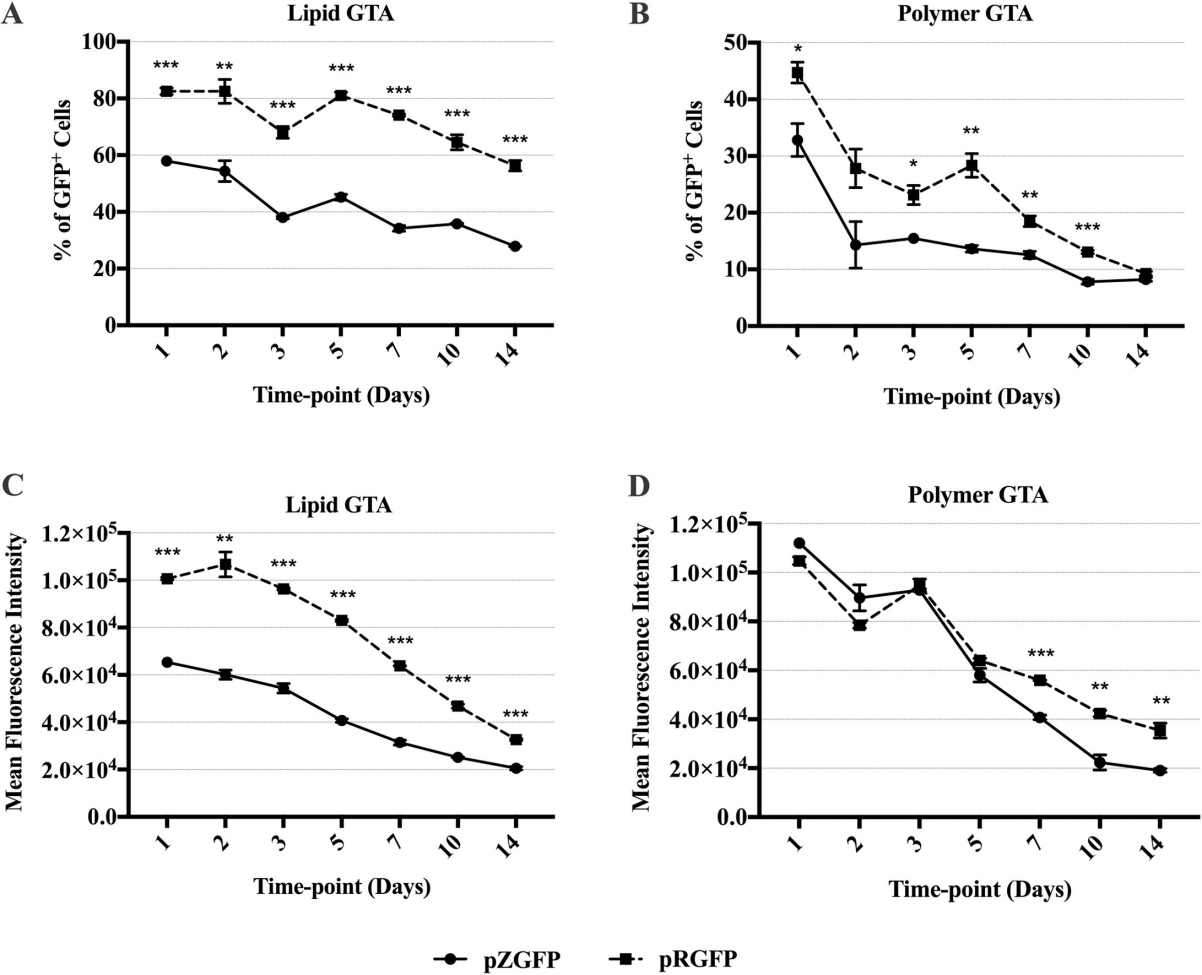
GFP expression of HEK-293FT cells transfected with either pZGFP (0 CpG) or pRGFP (60 CpGs), complexed with Lipid or Polymer GTA. A comparison of GFP expression between pZGFP (solid line) and pRGFP (dashed line) was assessed in terms of % of GFP^+^ cells, (A, B), and mean fluorescent intensity (MFI), (C, D). GFP expression was measured at Day 1, 2, 3, 5, 7, 10, and 14 post-transfection. Data are presented as mean ± S.E.M. of 3 biological replicates. Asterisk (*) indicates a significant difference for a given time-point between pCpG-free and pCpG-rich determined by Student’s t-test (*p<0.05, **p<0.01, ***p<0.001).

To determine the effects of transgene CpG depletion on gene expression, the pZGFP (0 CpG) and pRGFP (60 CpGs) were transfected into HEK-293FT. Transfection was performed using 2 gene transfer agents (GTA), Lipid and Polymer, to account for differences in pDNA delivery and release in the cells, which may influence transgene expression [37]. In both groups, the percentage of GFP-expressing cells and the mean fluorescence intensity (MFI) (Fig 4A–4D) peaked at Day 1 post-transfection and declined steadily over the course of 14 days. At single cell level, Polymer GTA resulted in higher MFI than Lipid GTA for cells transfected with either pDNA.

Transfection using Lipid GTA resulted in the highest expression of pZGFP at Day 1 with 57.9% GFP^+^ cells, yet it was significantly lower (p<0.001) than pRGFP by 24.6% (Fig 4A). The percentage of GFP^+^ cells in the pZGFP-transfected group remained significantly lower than pRGFP up to the final time-point, where pZGFP exhibited lower GFP^+^ cells than pRGFP by 28.4% (p<0.001). As for the average single-cell expression, MFI of pZGFP^+^ cells was significantly and consistently lower compared to pRGFP from Day 1 (p<0.001) up to Day 14 (p<0.001) (Fig 4C). At the peak of expression on Day 1, MFI of pZGFP-transfected cells was ≈ 3.5 x 10^4^ lower than pRGFP. The difference became smaller by Day 14, but still at the order of 10^4^.

A similar trend was observed with Polymer GTA, where pZGFP exhibited a significantly lower percentage of GFP^+^ cells than pRGFP at all time points (Fig 4B). On the other hand, there was no significant difference in MFI at Day 1, 2, 3 and 5 between pZGFP and pRGFP groups (Fig 4D). However, with time, the MFI of pZGFP became significantly lower than pRGFP from Day 7 (p<0.001) to Day 14 (p<0.01). Overall, the pZGFP (0 CpG) exhibited reduced reporter gene expression at the population and single-cell levels when compared to pRGFP (60 CpGs in transgene region) in HEK-293FT, regardless of GTA used.

### Reduced expression of pZGFP was not influenced by cell species and origin

**Fig 5:**
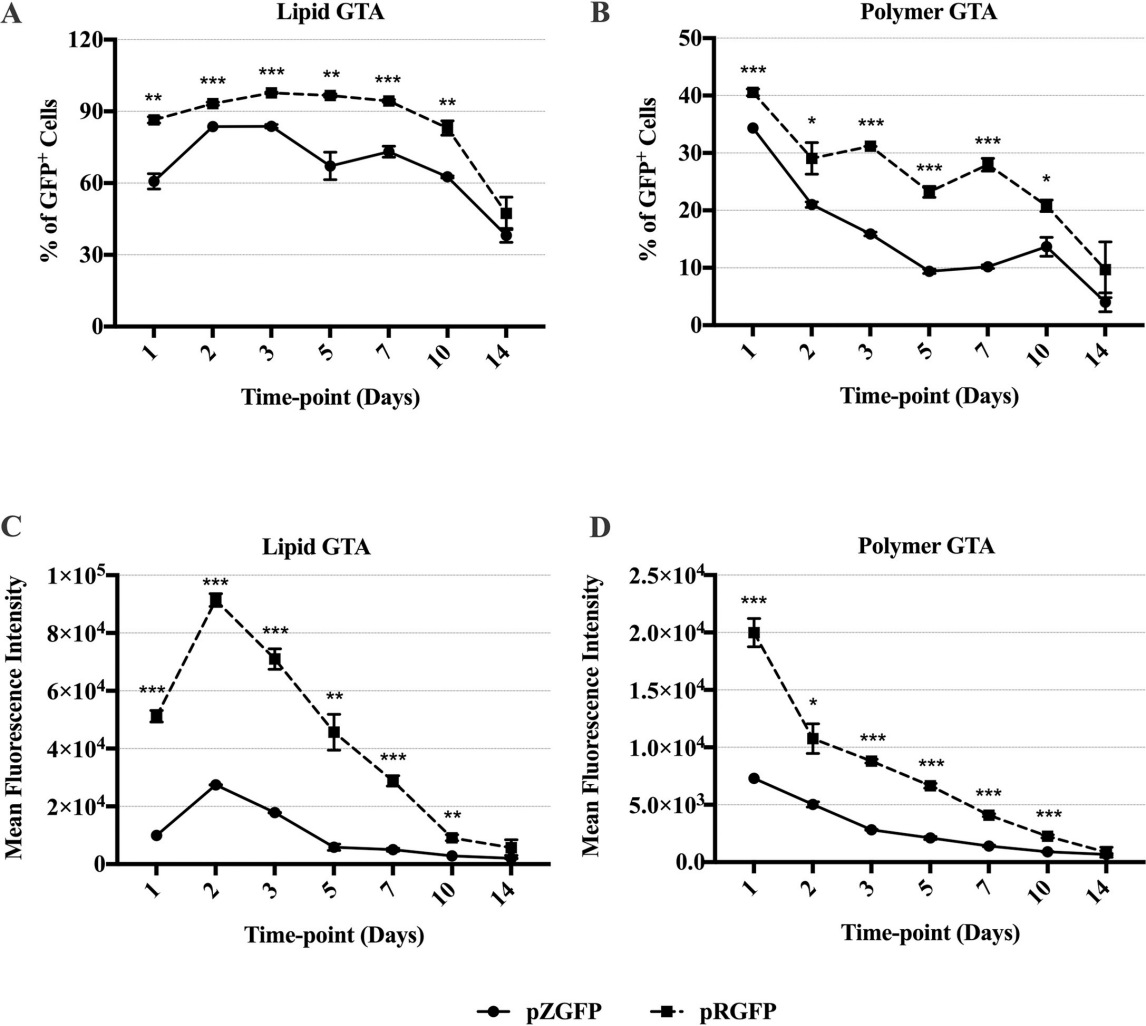
GFP expression of NIH-3T3 cells transfected with either pZGFP (0 CpG) or pRGFP (60 CpGs), complexed with Lipid or Polymer GTA. A comparison of GFP expression between pZGFP (solid line) and pRGFP (dashed line) was assessed in terms of % of GFP^+^ cells, (A, B), and mean fluorescent intensity (MFI), (C, D). GFP expression was measured at Day 1, 2, 3, 5, 7, 10, and 14 post-transfection. Data are presented as mean ± S.E.M. of 3 biological replicates. Asterisk (*) indicates a significant difference for a given time-point between pZGFP and pRGFP determined by Student’s t-test (*p<0.05, **p<0.01, ***p<0.001).

Expression of a transgene was shown to vary in different species as cell species influenced promoter activity, including the EF1α promoter [38], which was utilized in this study. Hence, to determine if the reduced pZGFP expression was species-specific, NIH-3T3 mouse cell line was transfected with pZGFP and pRGFP complexed with Lipid or Polymer GTA. Generally, with Lipid GTA, the percentage of GFP^+^ cells was ≈ 2 folds higher (pZGFP: 60.8%; pRGFP: 86.5%) than Polymer GTA (pZGFP: 34.4%; pRGFP: 40.6%). At Day 1, the group transfected with pZGFP exhibited significantly lower GFP^+^ cells (60.8%) compared to pRGFP (86.5%) when delivered using Lipid GTA (p<0.01). pZGFP-transfected group exhibited the highest percentage of GFP^+^ cells at Day 2 (83.6%) but was still significantly lower (p<0.001) than pRGFP (97.7%). The percentage of GFP^+^ cells from pZGFP transfected group remained lower than pRGFP-transfected group at all time points, except at Day 14. A similar trend was also observed with polymer GTA (Fig 5A and 5B). Mean fluorescence intensity was also significantly lower in the pZGFP-transfected group at all time points except at Day 14, regardless of GTA used (Fig 5C and 5D).

**Fig 6:**
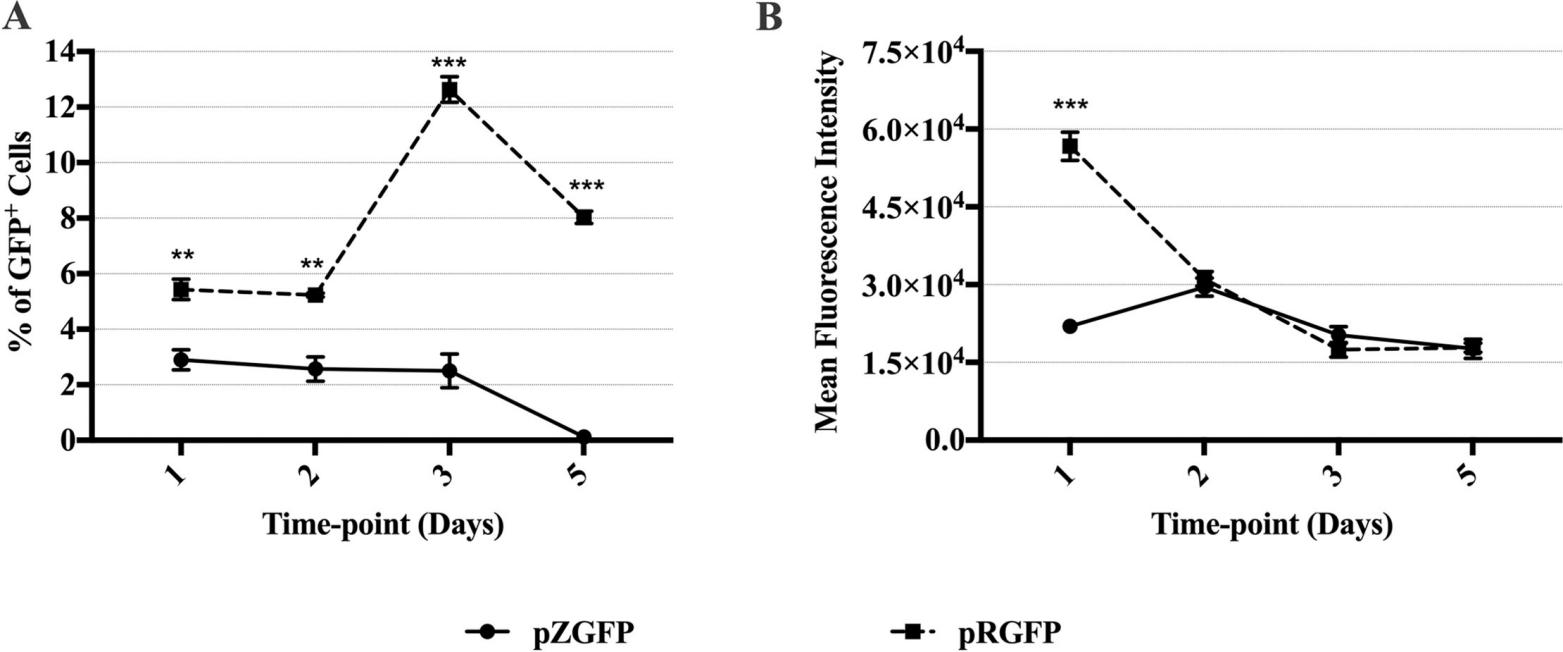
GFP expression of primary mouse fibroblast cells transfected with either pZGFP (0 CpG) or pRGFP (60 CpGs) complexed with Lipid GTA. Comparison of GFP expression between pZGFP (solid line) and pRGFP (dashed line) was assessed in terms of % of GFP^+^ cells, (A) and mean fluorescent intensity (MFI) (B). GFP expression was measured at Day 1, 2, 3, 5, 7, 10, and 14 post-transfection. Data are presented as mean ± S.E.M. of 3 biological replicates. Asterisk (*) indicates a significant difference for a given time-point between pZGFP and pRGFP determined by Student’s t-test (*p<0.05, **p<0.01, ***p<0.001).

Since primary cells are better at simulating *in vivo* gene expression conditions as compared to immortalized cell lines [39, 40], mouse primary fibroblast cells were utilized to assess pZGFP and pRGFP expression profiles. Due to technical challenges and early cell senescence, GFP expression was only assessed at Day 1, 2, 3, and 5 post-transfection using Lipid GTA. The % of GFP^+^ cells obtained at Day 1 with Lipid GTA was minimal, with pZGFP 2.5% lower than pRGFP (p<0.01) (Fig 6A). Despite the low % of GFP^+^ cells, the inferior expression of pZGFP compared to pRGFP was still significant. The expression of pZGFP remained at a similar level up to Day 3 before receding to a negligible value at Day 5. Conversely, increased % of GFP^+^ cells was observed from pRGFP at Day 3 (12.6%), and the expression remained higher than pZGFP at the last time-point (p<0.01) (Fig 6A). Interestingly, the low expression of pZGFP at Day 1 was also reflected at the single-cell level, as the MFI value of pZGFP was ≈ 2.8 fold lower than pRGFP (p<0.001) (Fig 6B).

Here, pZGFP expression was significantly lower than pRGFP in both NIH-3T3 mouse cell line and primary mouse fibroblasts cells. This observation, supplemented with previous experiments, demonstrates that the absence of CpG in the transgene of pZGFP resulted in lower expression in *in vitro* conditions.

### pZGFP expression was not compromised by cytotoxicity

**Fig 7:**
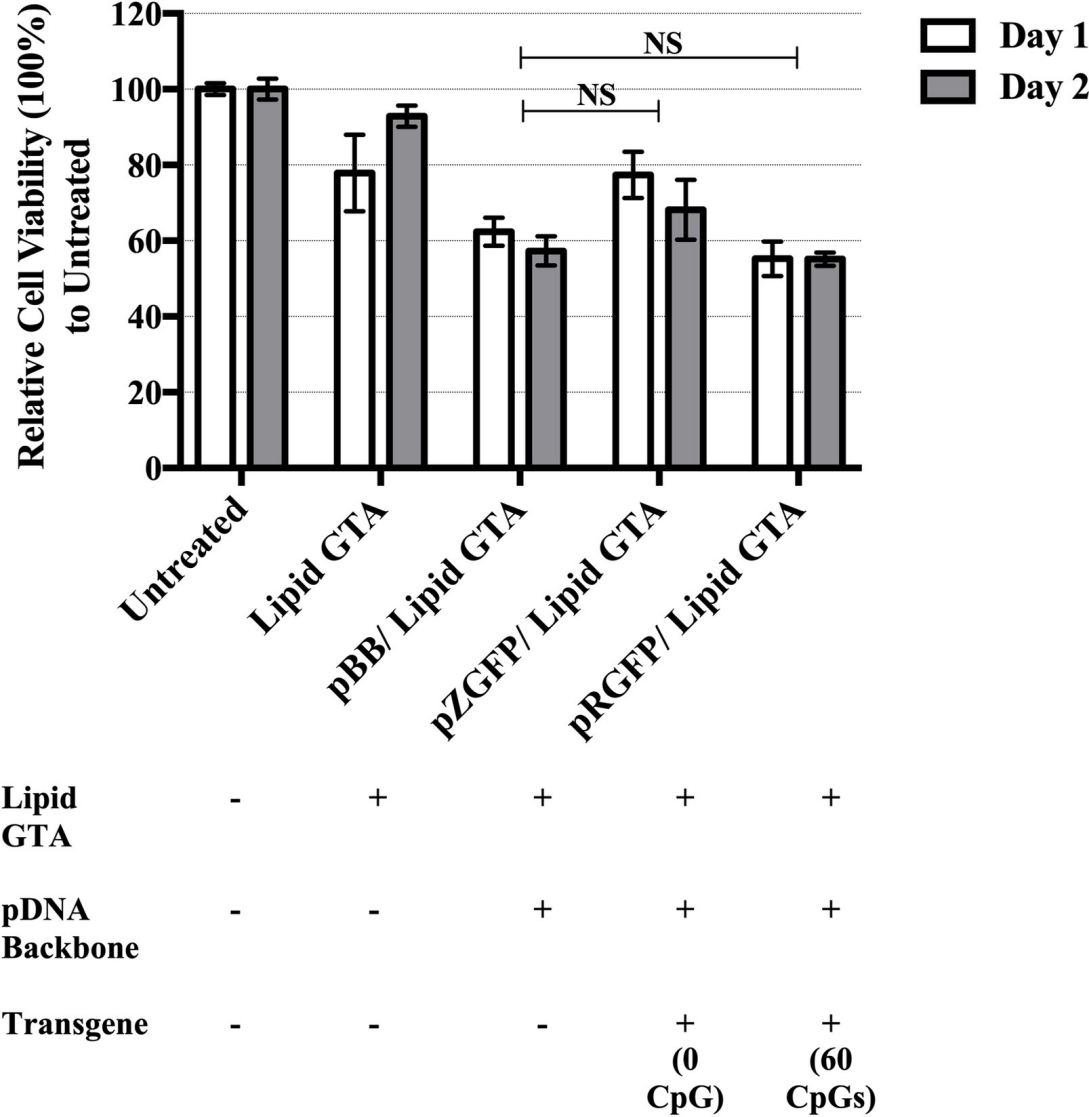
Cell viability analysis of HEK-293FT transfected using pZGFP or pRGFP complexed with Lipid GTA. The toxicity of pZGFP and pRGFP was assessed on HEK-293FT cells at Day 1 (white bars) and Day 2 (grey bars) post-transfection. pBB refers to a circularized CpG-free plasmid backbone (without transgene) identical to pZGFP and pRGFP. “-”sign indicates the exclusion, and “+” indicates the inclusion of the experimental component during transfection at Day 0. Data are presented as mean ± S.E.M. of 3 biological replicates. NS indicates no significant difference determined by Student’s t-test (*p<0.05).

Sequence-specific toxicity was observed where non-coding viral sequences are more toxic than non-coding eukaryotic sequences [41]. Since some viruses have CpG-depleted genomes [42], complete CpG-depletion in pZGFP may appear as an infection signal to the cells, leading to the cytotoxicity of pZGFP-transfected cells. To ascertain if the low expression of pZGFP in normal HEK-293FT cell line was not due to increased cytotoxicity effect, cell viability was evaluated upon exposure to the pDNA-GTA complexes. Cells were also transfected with a circularized pCpG-free backbone (pBB) complexed with Lipid GTA as a control (Fig 7). At Day 1, the cell viability from Lipid GTA only control group was slightly reduced. When complexed with the pDNAs, cell viability was reduced even further, to as low as 55.2% in the pRGFP/Lipid GTA group. Importantly the results indicate that pZGFP/Lipid GTA (77.3%) did not exhibit increased toxicity compared to pRGFP/Lipid GTA (55.2%). To rule out the possibility if pZGFP had any latent toxic effects, the cytotoxicity effect was further analyzed at Day 2. Interestingly at Day 2, pZGFP/Lipid GTA did not exhibit increased toxicity compared to pRGFP/Lipid (68.1% versus 55.1%, Fig 7).

In addition, the effect of transgene CpG depletion on cytotoxicity was also investigated. This was analyzed by comparing the cell viability of pZGFP/Lipid and pRGFP/Lipid-transfected cells to cells transfected with pCpG-free backbone (without transgene)/Lipid GTA. At Day 1, the difference was not significant between pBB (62.3%) and pZGFP (77.3%) or pRGFP (55.2%). Similarly at Day 2, the difference was not significant between pBB (57.3%) and pZGFP (68.1%) or pRGFP (55.1%). These data indicate that the absence of CpG motif on transgene in pZGFP did not cause any evident cytotoxicity leading to the lower expression.

### Comparable pZGFP and pRGFP gene dosages delivered to cells

**Fig 8:**
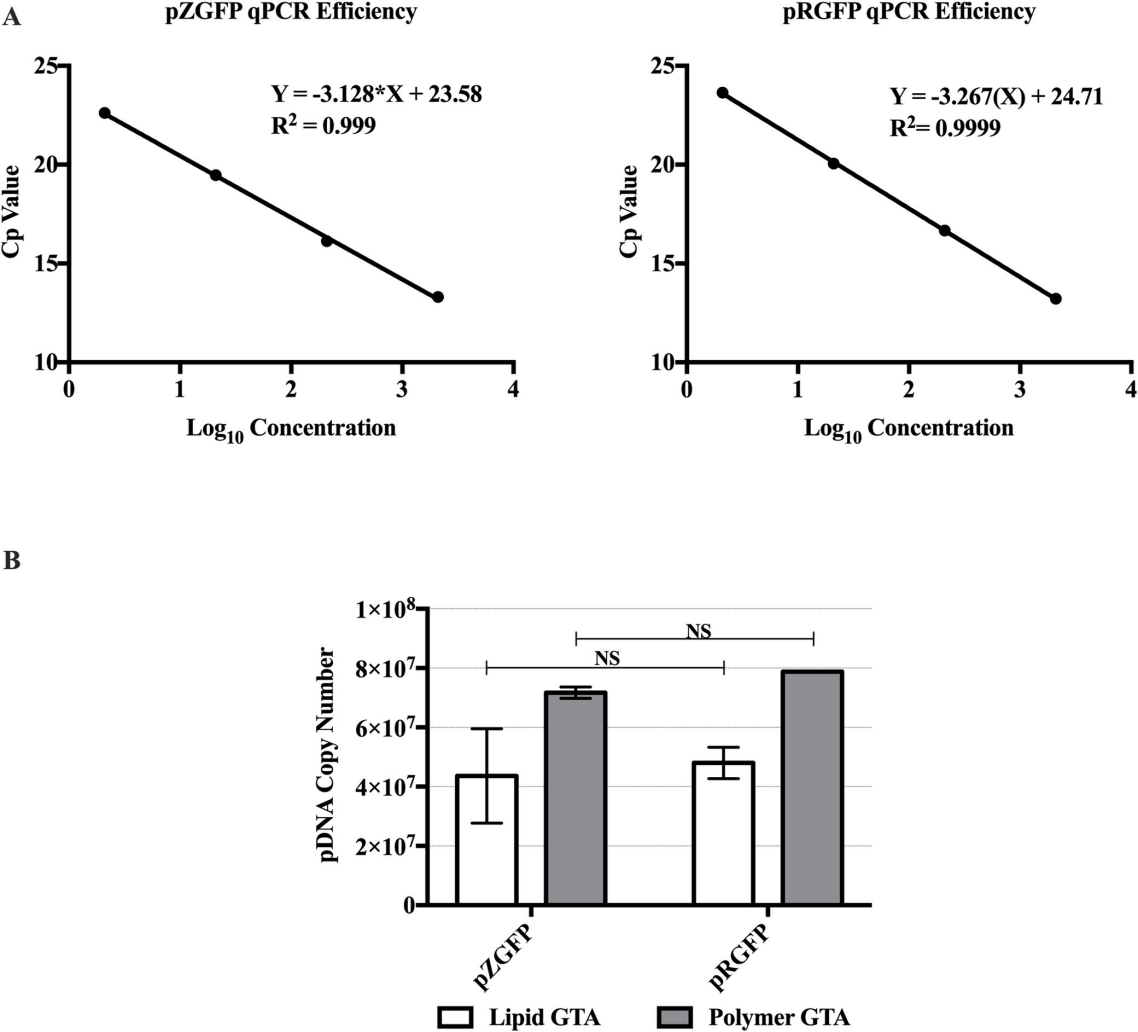
Gene dosage analysis of pZGFP and pRGFP at Day 1 post-transfection using Lipid and Polymer GTA. (A) qPCR efficiency for pZGFP and pRGFP were determined using serially diluted purified plasmids from 210 ng to 21 pg. (B) Plasmid copy numbers from HEK-293FT cells transfected with either pZGFP or pRGFP at Day 1 post-transfection. Data are presented as mean ± S.E.M. of 3 biological replicates. NS indicates no significant difference between pZGFP and pRGFP for each GTA, determined by Student’s t-test (*p<0.05).

Since the difference in expression between pZGFP and pRGFP began at Day 1, one may postulate that reduced gene dosage of pZGFP contributed to the low expression of pDNA. To determine the difference in gene dosage between pZGFP and pRGFP-transfected cells, plasmid copy number was quantified using absolute qPCR. Total DNA extracted from cells at Day 1, which were transfected with the pDNAs using Lipid or Polymer GTA, were utilized as input for qPCR. The qPCR standard curves were generated using known concentrations of purified pZGFP and pRGFP. The PCR efficiency and R^2^ values for pZGFP standard curve were 108.8% and 0.999, and for pRGFP standard curve were 102.3% and 0.999 (Fig 8A). These values were within acceptable range (PCR efficiency: 90–110%; R^2^ > 0.985) and indicate the qPCR assays were efficient. Based on the copy numbers from the whole cell population, Polymer GTA (pZGFP: 7.17 x 10^7^ copies, pRGFP: 7.88 x 10^7^ copies) was able to deliver ≈ 1.5 fold higher pDNAs compared with Lipid GTA (pZGFP: 4.36 x 10^7^ copies, pRGFP: 4.8 x 10^7^ copies). However, comparing pZGFP to pRGFP within each GTA, there was no significant difference in terms of pDNA copy number (Fig 8B), indicating that the limited expression of pZGFP was not due to the difference in gene dosage when compared to pRGFP.

### Expression of pZGFP was restricted at the transcriptional level, not by mRNA export

**Fig 9:**
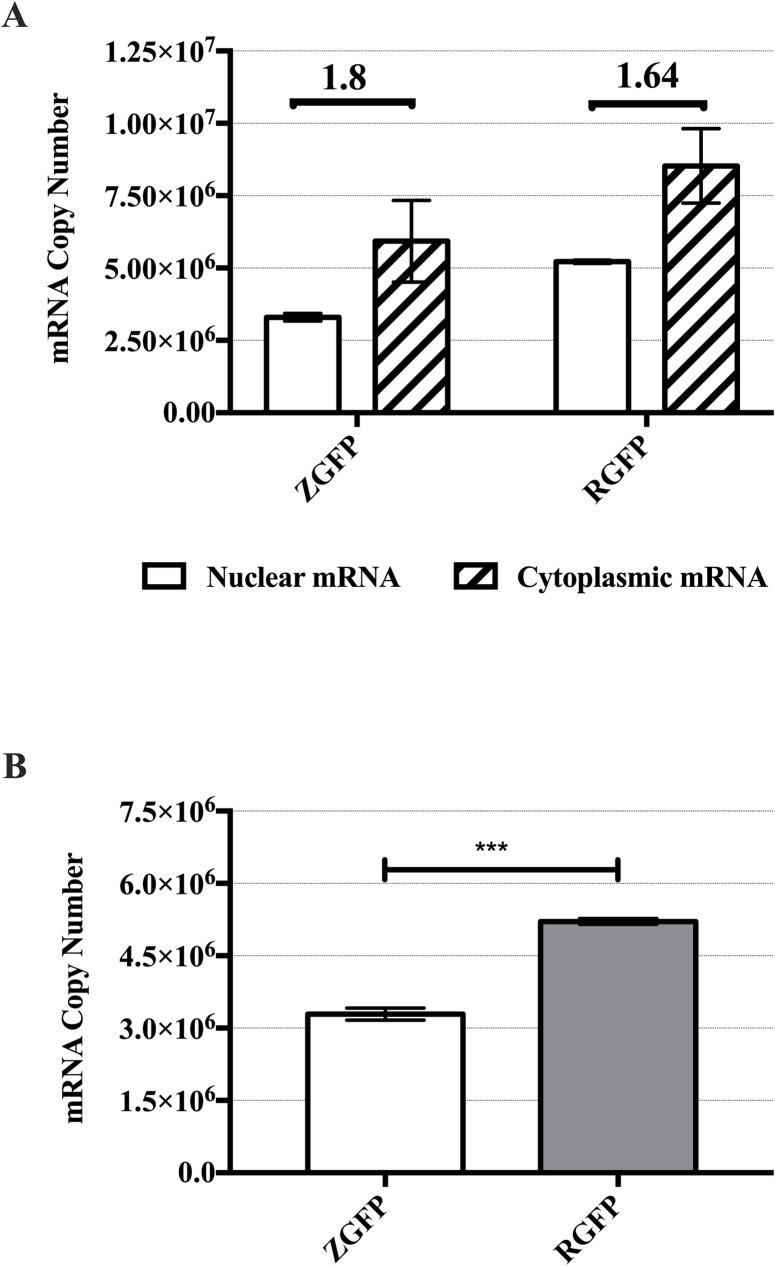
GFP mRNA distribution analysis of pZGFP and pRGFP at Day 1 post-transfection. mRNA copy numbers from HEK-293FT cells transfected with either pZGFP or pRGFP transfected at Day 1 post-transfection using Lipid GTA. (A) mRNA export rate (ratio of cytoplasmic mRNA copy number/ nuclear mRNA copy number) for pZGFP and pRGFP transfected cells were 1.80 and 1.64. White bars: nuclear mRNA; striped bars: cytoplasmic mRNA. (B) The difference in the steady state nuclear mRNA levels at Day 1 post-transfection was highlighted by plotting the nuclear fraction of transgenes extracted from Fig 9A. Data are presented as mean ± S.E.M. of 3 biological replicates. Asterisk (*) indicates a significant difference between ZGFP and RGFP determined by Student’s t-test (***p<0.001).

5-methylcytosine (m^5^C) is a post-transcriptional modification that is enriched in CpG-rich regions of the mRNA to promote mRNA export for translation [43]. To elucidate if the limited expression of CpG-depleted pZGFP was due to nuclear retention of *ZGFP* mRNA, nuclear and cytoplasmic mRNA of transfected cells at Day 1 were quantified using absolute RT-qPCR. The ratio of cytoplasmic_mRNA_/ nuclear_mRNA_ (eg. *ZGFP* cytoplasmic_mRNA_/ *ZGFP* nuclear_mRNA_) was used to indicate the mRNA export rate from the nucleus to the cytoplasm. Ratio value > 1 indicates more mRNAs are exported and < 1 indicates increased retention of mRNAs in the nucleus. Both pZGFP and pRGFP gave ratio value > 1 (pZGFP: 1.80; pRGFP: 1.64) (Fig 9A), demonstrating that the transcribed mRNAs were exported towards the cytoplasm instead of being retained in the nucleus. There was also no significant difference in the export rate, indicating that the mRNA export rate was similar, and the limited expression of pZGFP was not due to the *ZGFP* mRNA retention in the nucleus.

Using the same data set, the differences of transcript levels between nuclear fraction were further explored. The total mRNA of *ZGFP* (nuclear_mRNA_ + cytoplasmic_mRNA_ = ≈ 9 x 10^6^ mRNA copies) was observed to be much lower than total mRNA of *RGFP* (≈ 1 x 10^7^ mRNA copies). Notably, nuclear_mRNA_ of *ZGFP* (≈ 3 x 10^6^ mRNA copies) was significantly lower (p<0.01) than nuclear_mRNA_ *RGFP* (≈ 5 x 10^6^ mRNA copies) (Fig 9B). This signifies that the levels of steady state mRNA of pZGFP was lower than pRGFP.

### Increased nucleosome enrichment in pZGFP as compared to pRGFP

**Fig 10:**
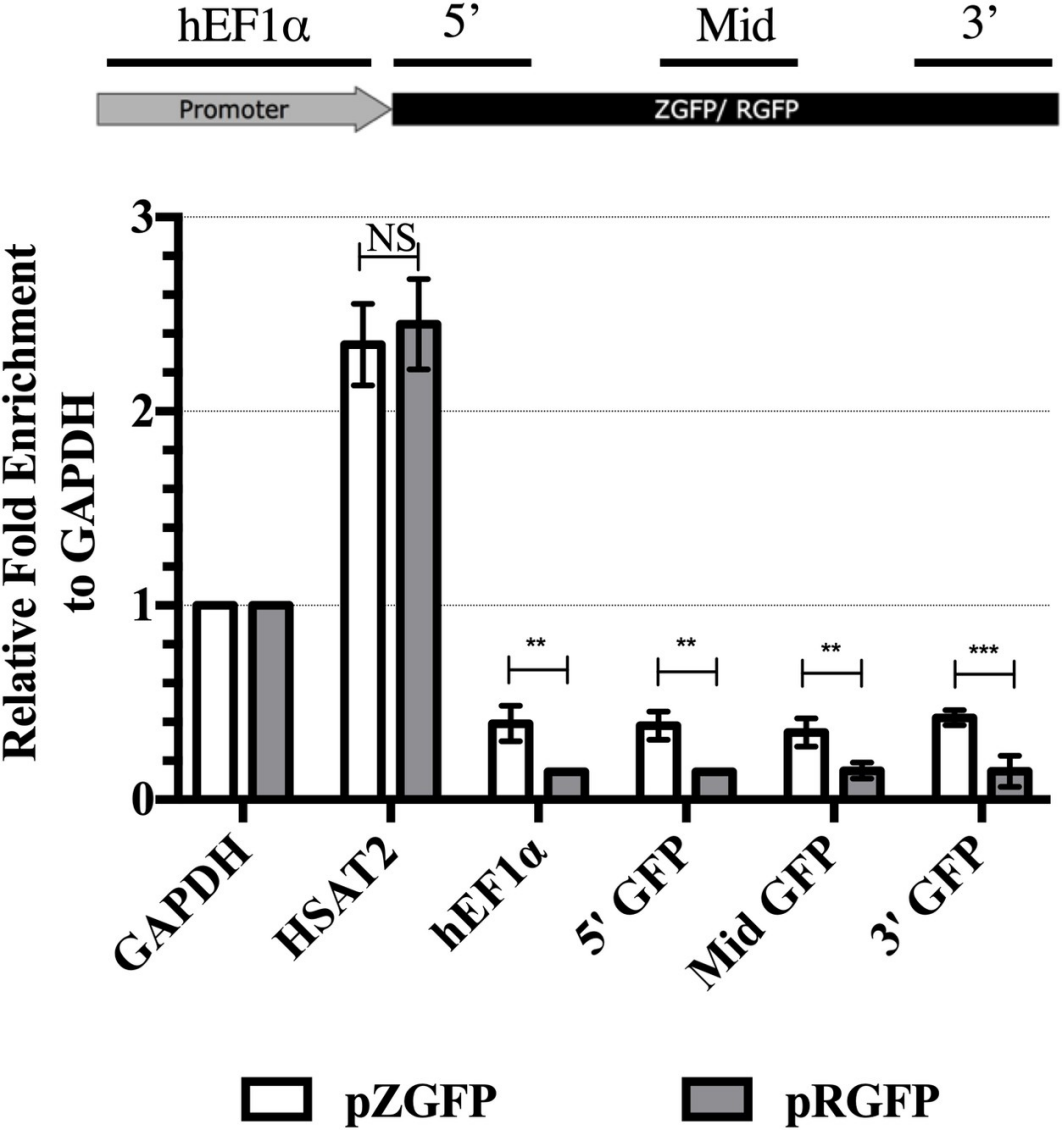
Nucleosome enrichment of pZGFP and pRGFP at Day 1 post-transfection. FAIRE-qPCR was performed on total DNA extracted from transfected cells. qPCR was targeted at the regions of promoter, 5’, middle and 3’ of the transgenes. GAPDH and HSAT2 are internal controls for nucleosome depleted and nucleosome enriched regions. Data are presented as mean ± S.E.M. of 3 biological replicates. Asterisk (*) indicates a significant difference for a given time-point between pZGFP and pRGFP determined by Student’s t-test (**p<0.01, ***p<0.001).

Nucleosome enrichment in the promoter or coding sequences can result in the repression of gene expression [44]. To determine if the reduction of pZGFP mRNA levels was due to nucleosome enrichment, FAIRE-qPCR was performed on HEK-293FT cells transfected with non-integrating pZGFP or pRGFP complexed with Lipid GTA at Day 1. FAIRE involves the non-specific cross-linking of histones to the DNA, fragmentation the cross-linked DNA, isolation by phenol-chloroform extraction and finally quantification of selected regions by qPCR. Human Satellite 2 (*HSAT2*) and *GAPDH* were used as internal controls. *HSAT2* is a satellite repeat sequence found at the centromeric regions and known to be heterochromatic region with increased nucleosome enrichment [45]. Similar fold of nucleosome enrichment of 2.34 and 2.44 in HSAT2 region was observed from pZGFP and pRGFP-transfected cells, respectively (Fig 10). This indicates that these internal control regions were highly chromatinized and the FAIRE experiment was successful. Compared to the housekeeping gene *GAPDH*, nucleosome density was low in overall regions of pZGFP (0 CpG) and pRGFP (60 CpGs) with the average of ≈ 0.4 fold and ≈ 0.15 fold, respectively. This was expected with constitutive transgene expression from abundant pDNA copies. Interestingly, transgene regions of pZGFP were consistently enriched with nucleosomes as compared to pRGFP as the average fold change difference was ≈ 2.7 fold. In addition, the *hEF1α* promoter in pZGFP has higher nucleosome density by 2.8 fold compared to the *hEF1α* promoter region in pRGFP, despite both having identical CpG-free hEF1α promoter. Overall, these results show that pZGFP (0 CpG) has higher nucleosome density than pRGFP (60 CpGs).

## Discussion

CpG-free pDNA has been reported to be an effective vector for transgene expression *in vivo* [6, 20, 22–25, 27] and was valuable in gene therapy clinical trial [46]. Despite the wealth of *in vivo* studies, a detailed assessment of CpG-free pDNA in *in vitro* systems has not yet been reported. Understanding the *in vitro* expression profile of CpG-free pDNA is beneficial as sustained ectopic expression could be achieved with CpG-free pDNA, which is not susceptible to DNA methylation-mediated transgene-silencing [15, 17, 47, 48]. Hence, in this study, we first sought to compare the expression levels of pCpG-free and pCpG-rich in human cell lines. We hypothesized that pCpG-free would provide sustained transgene expression as it was devoid of CpG dinucleotides and carries the eukaryotic EF1α promoter, as compared with pCpG-rich that would exhibit poor expression due to the presence of silencing-prone CpGs and viral CMV promoter. The cell lines tested include H1299 to represent lung cells because the CpG-free pDNA was extensively validated in the lungs *in vivo* [6, 19, 22, 23]. Surprisingly, in contrast to *in vivo* studies, pCpG-free expression was consistently lower than pCpG-rich during the 14 days course in the human cell lines (Fig 2).

The difference in expression level between the *in vivo* and *in vitro* systems might be explained by the inflammatory response evoked by the pDNA. In an *in vivo* environment, CpG-containing pDNA interacts with Toll-like Receptor 9 (TLR9) to induce an inflammatory response [49, 50]. As a result, activation of 1) Th1-type adaptive immune response [51] leading to the destruction of transgene-carrying cells and loss of transgene expression [7, 8], and 2) pro-inflammatory cytokines, such as TNF-α and IFN-γ, repress transgene expression, regardless of viral or mammalian promoter usage [10]. Therefore, CpG-induced inflammation *in vivo* is a major barrier to transgene expression, unlike in *in vitro* system. Here, pCpG-rich that contains 317 CpGs displayed more robust transgene expression capabilities than pCpG-free in the non-immunological *in vitro* models that lack pro-inflammatory response. This has led us to question the relevance of CpG depletion from transgene for enhanced or sustained *in vitro* expression. Noteworthy, the presence of CpG in the transgene region has been shown to lead to a higher *in vitro* transgene expression compared to CpG-depleted transgene [52]. Unlike non-integrating pDNAs used in our study, Krinner et al. (2014) utilized an integrating system called Flp—In™, where the integrated transgene facilitates constitutive expression. Integration of transgene in the host genome has been reported to cause epigenome perturbations and unlikely to provide “actual” gene expression outcomes [53]. Moreover, the expression dynamics between non-integrating vector, such as CpG-free pDNA, and integrating vector differ significantly [54]. Therefore, further investigation is required to understand the effects of CpG content on transgene expression in the context of non-integrating pDNA.

The pCpG-free and pCpG-rich pDNA constructs used in the earlier study had different pDNA backbones (*i*.*e*. EF1α promoter vs CMV promoter) and was not suitable to investigate the influence of transgene CpG content on expression. Therefore, we constructed pDNAs of identical CpG-free backbone with CpG-depleted transgene (pZGFP; 0 CpG) or CpG-containing transgene (pRGFP; 60 CpG). Efforts have been made to ensure that any transcriptional variability was due only to the CpG content of the transgene, and not to any other elements in the pDNA construct (Fig 3). First, these plasmids had identical regulatory elements (*i*.*e*. enhancer, promoter, introns, polyA). Next, the scaffold/ matrix attachment region (S/MAR) was included to prevent gene silencing [2] caused by transcriptional interference from the encroachment of heterochromatin from the backbone [55]. Lastly, humanized CpG-depleted allele (*ZGFP*; 0 CpG) and CpG-containing GFP allele (*RGFP*; 60 CpG) were selected to determine the influence of CpG depletion from transgene. These alleles encoded proteins that was 98% identical with GFP fluorophore (Leu_65_-Thr_66_-Tyr_67_) to ensure no difference in the ZGFP and RGFP ability to fluoresce that would influence protein expression analysis [36].

Next, we systematically evaluated factors that would impact transgene expression leading to the limited expression of pZGFP, as compared with pRGFP. Regardless of distinct GTAs used, the pZGFP expression was consistently low in the human cell line, mouse cell line, and primary cells (Figs 4–6), indicating that the abated pZGFP expression was not due to the discrepancy in pDNA delivery or cell types. There was also no discernable cytotoxicity detected that might have caused the loss of pZGFP-transfected cells (Fig 7). In addition, pDNA copy number analysis revealed comparable gene dosage in both pZGFP and pRGF-transfected cells (Fig 8). These data signify the implication of CpG-depletion on transgene expression, as the only difference between the two pDNAs was the variability in the number of CpG motifs in the transgene. This observation coincides with Krinner *et al*. (2014), where integrated CpG-depleted transgene in chinese hamster ovary (CHO) cells exhibited consistently low expression compared to CpG-containing transgene up to 2 years [52]. The inferior *GFP* activity from pZGFP, despite having similar gene dosage with pRGFP, implies the involvement of a mechanism downstream of gene dosage. Subsequently, we discovered that the total *ZGFP* mRNA was significantly lower than total *RGFP* mRNA, clearly indicating a reduced transcription of pZGFP with CpG-depleted transgene (Fig 9). Given the identical enhancer/promoter and the backbone in pZGFP and pRGFP, the transcription activity of these pDNAs was most probably regulated by any of the epigenetic mechanisms that modulate regulatory element activity [56]. However, a limitation of this study is that epigenetic regulation may not be the sole cause for low pZGFP expression. Other possible confounding factors, such as mRNA secondary structures, RNA decay, translational efficiency, and miRNA cryptic sites [57], have not been investigated in this study.

The formation of condensed or loosed chromatin structure by nucleosome occupation affects the accessibility of transcription factors and transcription initiation complexes that dictate the levels of steady state mRNA [44]. FAIRE-qPCR analysis in our study revealed that pZGFP had higher nucleosome density at the promoter and transgene regions than the pRGFP (Fig 10). This highly indicates that the reduced levels of steady state mRNA in pZGFP is associated with the increase in nucleosome enrichment. Interestingly, earlier studies using micrococcal nuclease (MNase) digestion of nuclei and Southern hybridization have shown that transfected pDNA form nucleosomes in mammalian, despite being independent of the host genome [58–60]. As a result, pDNA expression could be improved or restricted by depleting or increasing nucleosome density on pDNA [58, 61]. In addition, studies have shown a strong correlation between CpG and nucleosome density. Global analysis of CpG-depleted genes in mouse and human revealed these regions were insensitive to DNAse I and have increased chromatin inaccessibility [62]. In the context of CpG-depleted transgene, Krinner *et al*. (2014) reported that integrated CpG-depleted transgene exhibited lower expression levels due to increased nucleosomes within the transgene, where else integrated CpG-containing transgene was able to provide expression at higher levels due to nucleosome displacement [52]. To date, no study has shown the nucleosome enrichment as the limiting factor for CpG-free pDNA expression and its mechanism. One possible explanation is the complete CpG depletion in pZGFP may have affected the physical properties of the pDNA. The presence of CpG dinucleotides was shown to affect DNA curvature and bendability, where adding more CpGs further reduce hydrodynamic radius and limit the nucleosome occupation in the DNA [63]. The FAIRE-qPCR technique used in this study, however, only provided general nucleosome density within a DNA region, and higher resolution mapping techniques, such as MNase-Seq, would provide detailed insight into pZGFP-nucleosome interaction [64].

Our findings revealed a conundrum to CpG-free pDNA usage, as complete CpG depletion minimizes detrimental inflammation and avoids DNA methylation silencing but could also leads to limited expression due to increased nucleosome occupation. One potential solution is to include a left-handedly curved DNA sequence (TTTTT[CATGTTTTT]_3_) in CpG-free pDNA, as this sequence caused nucleosome sliding and lead to significant improvement of transgene expression in cell lines and mice [65, 66]. In summary, our data show that the CpG-free pDNA transgene expression *in vitro* was significantly reduced at the transcriptional level. This is most likely due to nucleosome enrichment that limits the transgene expression, and to date, this is the first report that suggests the impact of nucleosome density on non-integrating CpG-free pDNA expression.

## Abbreviations

CpG: cytosine guanine dinuclotide
pDNA: non-integrating plasmid DNA
pZGFP: CpG-free plasmid backbone containing CpG-free GFP
pRGFP: CpG-free plasmid backbone containing CpG-rich GFP
TLR9: Toll-like receptor 9
FAIRE: Formaldehyde-Assisted Isolation of Regulatory Elements
